# Single-Cell RNA Sequencing Identifies CCR6-Driven Immune Landscape Changes in RM1 Prostate Cancer Bone Metastasis

**DOI:** 10.1089/dcbr.2025.0001

**Published:** 2025-02-28

**Authors:** Marxa L. Figueiredo

**Affiliations:** 1 Department of Basic Medical Sciences, College of Veterinary Medicine, Purdue University, West Lafayette, IN 47907, USA

**Keywords:** single-cell RNA sequencing, immune cells, RM1 mouse model, prostate cancer, human bone metastatic

## Abstract

**Background:**

Prostate cancer commonly metastasizes to bone, creating an immunosuppressive microenvironment that supports tumor progression. The RM1 (Ras/Myc) mouse model is a valuable tool for studying interactions between prostate tumors and the bone marrow immune landscape.

**Methods:**

Single-cell RNA sequencing was employed to investigate how CCR6 deficiency affects immune cell comsmunication in bone marrow from RM1 prostate cancer bone metastasis. Immune responses were compared between wild-type and CCR6 knockout (CCR6ko) C57BL/6-mice injected with RM1-BoM3 cells.

**Results:**

Distinct immune cell dynamics were observed between the two groups. CCR6ko bone marrow showed enhanced signaling from macrophages, regulatory T cells, and myeloid-derived suppressor cell-like cells, with altered receptor activity in naïve T cells, NK cells, and conventional dendritic cells. Differential gene expression highlighted immune regulation pathways and transcriptional networks centered on JUN, JUNB, and FOSB, linked to immune suppression and tumor progression. Cell-cell communication analysis revealed changes in CCL, TGFb, MIF, and CXCL pathways, aligning with observations in human castration-resistant prostate cancer (CRPC) bone-metastasis. Profiling of immune cell populations showed increased tumor-infiltrating monocytic cells, M2 macrophages, and NKT-like CD8 cells in RM1 tumors compared to CCR6ko bone marrow. Pathway enrichment analysis identified upregulated pathways, including IL-17 signaling and osteoclast differentiation, associated with immune-modulation and inflammation. Gene regulatory networks in the RM1 bone metastasis microenvironment involved NFKB1, STAT1, IRF8, and JUN family proteins, emphasizing their roles in immune suppression and tumor progression.

**Conclusions:**

These results suggest that targeting CCR6 may enhance immunotherapy efficacy in prostate cancer bone metastasis by reshaping the immune microenvironment. Validation in human CRPC bone metastasis datasets revealed conserved immune interactions.

## Introduction

Prostate cancer is one of the most common malignancies in men and frequently metastasizes to bone, where it creates a highly complex microenvironment that promotes tumor growth and progression. Bone metastases in prostate cancer are particularly challenging to treat since they develop a highly immunosuppressive tumor microenvironment (TME) that limits effective antitumor immune responses.^1^ This altered microenvironment consists of tumor cells, stromal cells, and various immune cell populations, which may interact to either promote or inhibit tumor progression. Understanding the immune landscape at these metastatic sites is critical for developing novel therapeutic strategies aimed at reversing immune suppression and enhancing antitumor immunity.

Bone marrow is a key site of prostate cancer metastasis, where immune cell populations are profoundly altered, contributing to an immunosuppressive microenvironment that supports tumor growth.^2^ Despite the growing recognition of the importance of the immune system in modulating bone metastasis, the precise mechanisms by which immune cells communicate and adapt to the presence of metastatic prostate cancer remain poorly understood. The RM1 (Ras/Myc) mouse model of prostate cancer is widely used preclincally for studying how prostate tumors interact with and grow in the bone metastatic microenvironment.^3^ The model closely mimics the human disease in terms of tumor progression and immune cell infiltration, making it a promising tool for investigating tumor–immune interactions in bone metastasis.^4^

CCR6 (C-C Chemokine Receptor 6), a chemokine receptor expressed on several immune cell types, plays a crucial role in immune cell trafficking and has been implicated in various cancer models. In prostate cancer, CCR6 has been shown to influence the recruitment of specific immune cell subsets, including regulatory T cells (Tregs) and myeloid-derived suppressor cells (MDSCs), which are known to facilitate immune evasion.^5,6^ Previous studies have suggested that CCR6-CCL (Chemokine (C-C motif) ligand) signaling may contribute to the establishment of an immunosuppressive microenvironment in metastatic sites, but the detailed impact of CCR6 deficiency focusing on immune cell interactions in prostate cancer bone metastasis remains yet unclear.

In this study, we leveraged single-cell RNA sequencing (scRNA-seq), a powerful tool for dissecting the complexity of immune cell populations at a single-cell resolution, to analyze immune cell communication in the bone marrow microenvironment of RM1 prostate cancer bone metastasis. Using a dataset generated from bone marrow samples of wild-type (wt) and CCR6 knockout (CCR6ko) C57BL/6 mice implanted with RM1-BoM3 cells,^7^ a metastatic prostate cancer cell line derived from RM1 cells, we aimed to investigate how CCR6 deficiency impacts immune cell interactions and signaling pathways. By comparing immune responses in the bone marrow of wt and CCR6ko mice, we sought to understand how CCR6 inhibition might alter the immune landscape and reprogram tumor-immune cell communication. Our findings suggest that CCR6 knockdown or inhibition could be combined with novel pathways and combination therapies identified through cell–cell communication, gene network, and pathway analyses to enhance therapeutic efficacy. Additionally, the findings were validated in a human dataset from patients with bone metastatic castration resistant prostate cancer (bmCRPC).^7^ Ultimately, our goal is to uncover potential therapeutic strategies from these analyses that the field can use for modulating the bone marrow microenvironment in metastatic prostate cancer, with an emphasis on targeting immune cell signaling pathways to enhance antitumor immunity.

## Methods

### Samples

We performed scRNA-seq analysis from a dataset that sequenced bone marrow-derived samples isolated from mice implanted with RM1-BoM3 cells, a metastatic variant of the RM1 prostate cancer cell line. RM1-BoM3 cells were engineered to express TdTomato and luciferase for *in vivo* tracking and injected into the left ventricle of wt and CCR6ko C57BL/6 mice as described previously.^7^ Bone marrow was harvested, dissociated into single-cell suspensions, and sorted to enrich for immune cell populations using flow cytometry based on markers including CD45 to exclude non-hematopoietic cells. This ensured that only immune cells were subjected to scRNA-seq, as supported by the referenced source study. Data for this mouse study are publicly available under accession number GSE143791. For the human data, scRNA-seq datasets were obtained from a study deposited under the same accession number. Samples included normal bone marrow (7 benign and 8 distal, uninvolved bone marrow) and tumor-affected bone marrow (17 samples from patients with tumor-involved bone marrow).

### Bioinformatics analyses

#### Quality control and preprocessing.

Unfiltered count matrices were uploaded into the Trailmaker platform,^8^ where rigorous quality control was applied to ensure high-quality data. Cells with <500 unique transcripts were excluded to remove low-quality or empty droplets, while cells with more than 15% mitochondrial gene expression were eliminated to avoid including dead or dying cells. Potential doublets were identified and excluded using the scDblFinder method, resulting in the removal of ~5% of cells. Outliers exhibiting extreme RNA counts or aberrant gene expression profiles were filtered out using linear regression-based approaches. Following these steps, ~85% of the initial cells were retained for downstream analyses. Specifically, 12% of cells were excluded due to low transcript counts, 3% for high mitochondrial content, and 5% due to doublet identification. Data normalization, Principal Component Analysis, and integration with *Harmony* were performed on high-quality cells, for either the mouse dataset or the human dataset. Cluster-specific marker genes were identified through the Wilcoxon rank-sum test via the presto package.

#### Differentially expressed genes analysis.

We conducted *STRING-DB* analyses using the differentially expressed genes (DEGs) (*p* < 0.05) from either mouse [upregulated in the more malignant samples (RM1_bm)] relative to ccr6ko_RM1_bm samples. Also, we examined the human dataset upregulated DEGs in tumor versus benign bone marrow datasets to identify the cells and pathways that might be conserved or related across these two species.

#### Cell–cell communication and regulatory network analysis.

In *Icarus* (v3.0),^9^ clustering utilized the first 10 PCs with a k-nearest neighbor value of 10 and Louvain clustering at a resolution of 1.2, with UMAP (Uniform Manifold Approximation and Projection) for visualization (raster mode). For cell–cell communication analysis, we used CellChat to infer the intercellular communication patterns based on ligand–receptor interactions in scRNA-seq data. Gene regulatory networks were identified using SCENIC, integrating transcription factor binding motif enrichment and coexpression data. The SCENIC workflow included coexpression module detection with the GENIE3 algorithm, cis-regulatory motif analysis of gene promoters (up to 500 bp upstream and 20 kb around the transcription start site), and scoring regulon activity across cell clusters with the AUCell (Area Under the Curve Cell) method. By combining SCENIC and CellChat, we integrated transcriptional regulatory activity with cell–cell communication patterns to uncover key signaling pathways and regulatory networks that influence the immune microenvironment in prostate cancer bone metastasis. In summary, these methods incorporated complementary approaches, integrating ligand–receptor interactions and gene regulatory networks to enhance confidence in observed patterns.

## Results

### Shifts in immune cell populations, including tumor-infiltrating monocytic cells, M2 macrophages, and NKT-like cells, in the RM1 prostate cancer bone metastasis model

**FIG. 1.**
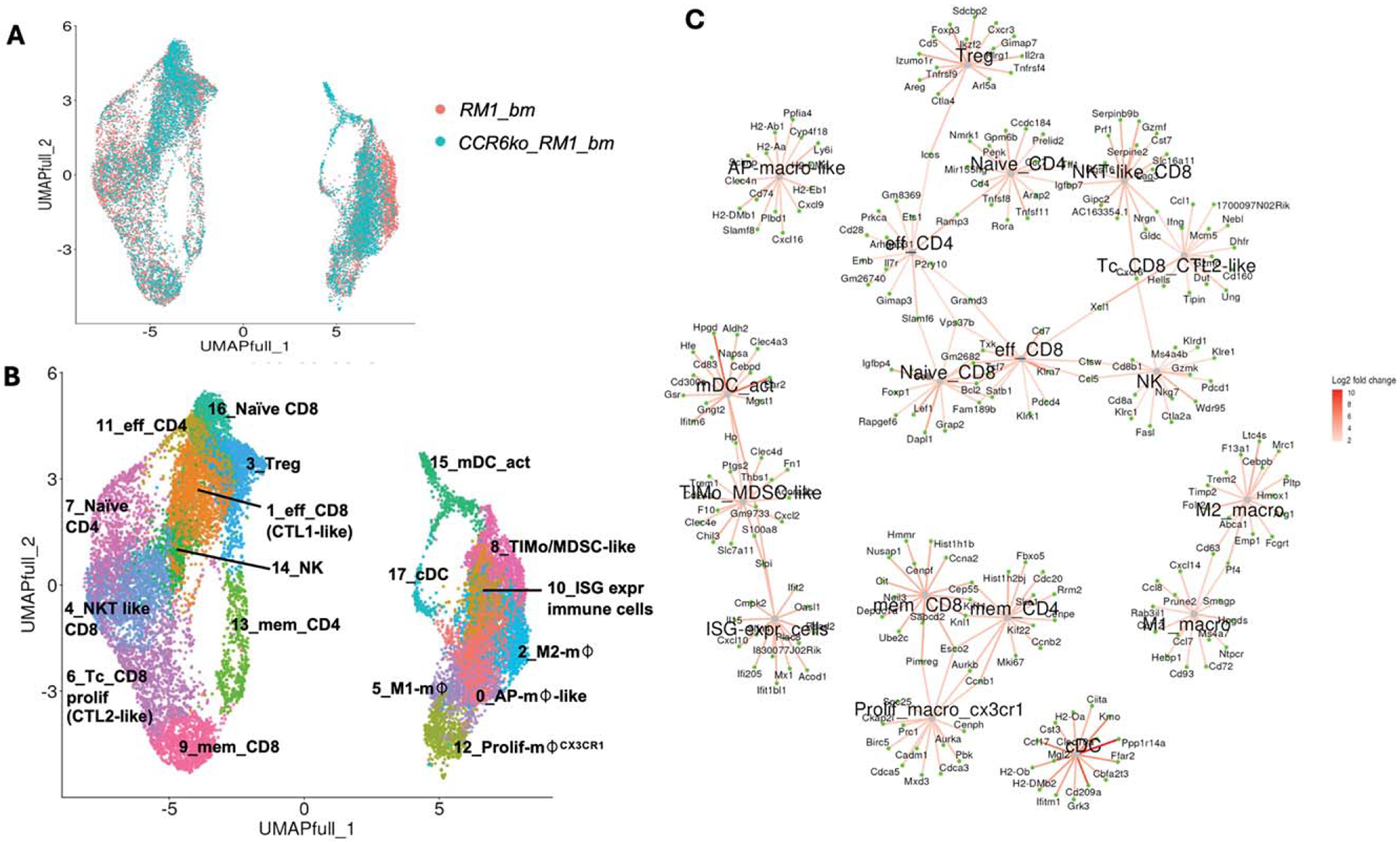
scRNA-seq of CD45+ cells from wild-type bone marrow bearing RM1 tumors (RM1_bm) or CCR6 knockout bone marrow bearing RM1 tumors (CCR6ko_RM1_bm). (a) UMAP of cellular changes comparing both samples. (b) Louvain clustering UMAP with cellular annotations for 17 unique clusters. (c) Marker gene map for the louvain clusters showing top genes in each cluster and the relationship between clusters with color bar (log2 fold change).

The findings from this study offer novel insights into the immune cell dynamics within the bone metastasis microenvironment of prostate cancer, specifically in RM1 tumors. Fig. 1 illustrates the immune cell populations identified from CD45+ sorted cells isolated from RM1 tumors growing in either wt or ccr6ko bone marrow, showing good data integration, with some differences visible in the overlap UMAP between the sample groups, including in the T cell subsets (left) and myeloid populations (right), with 17 clusters identified (Fig. 1a). The cell populations were subsequently annotated (Fig. 1b) based on subtype markers (Fig. 1c) and previously reported expression profiles.^7,10,11^ Cell types that were increased in quantity in the more malignant^7^ bone marrow tumor samples (RM1_bm) relative to the less malignant samples, ccr6ko bone marrow bearing RM1 tumors (ccr6ko_RM1_bm), included tumor-infiltrating monocytic cells (TIMo), which had markers similar to MDSC (MDSC-like) and M2 macrophages, as well as NKT (Natural Killer T)-like CD8 and naïve CD4 cells.

### Altered cell–cell communication and pathway signaling in ccr6ko bone marrow in RM1 prostate cancer model

**FIG. 2.**
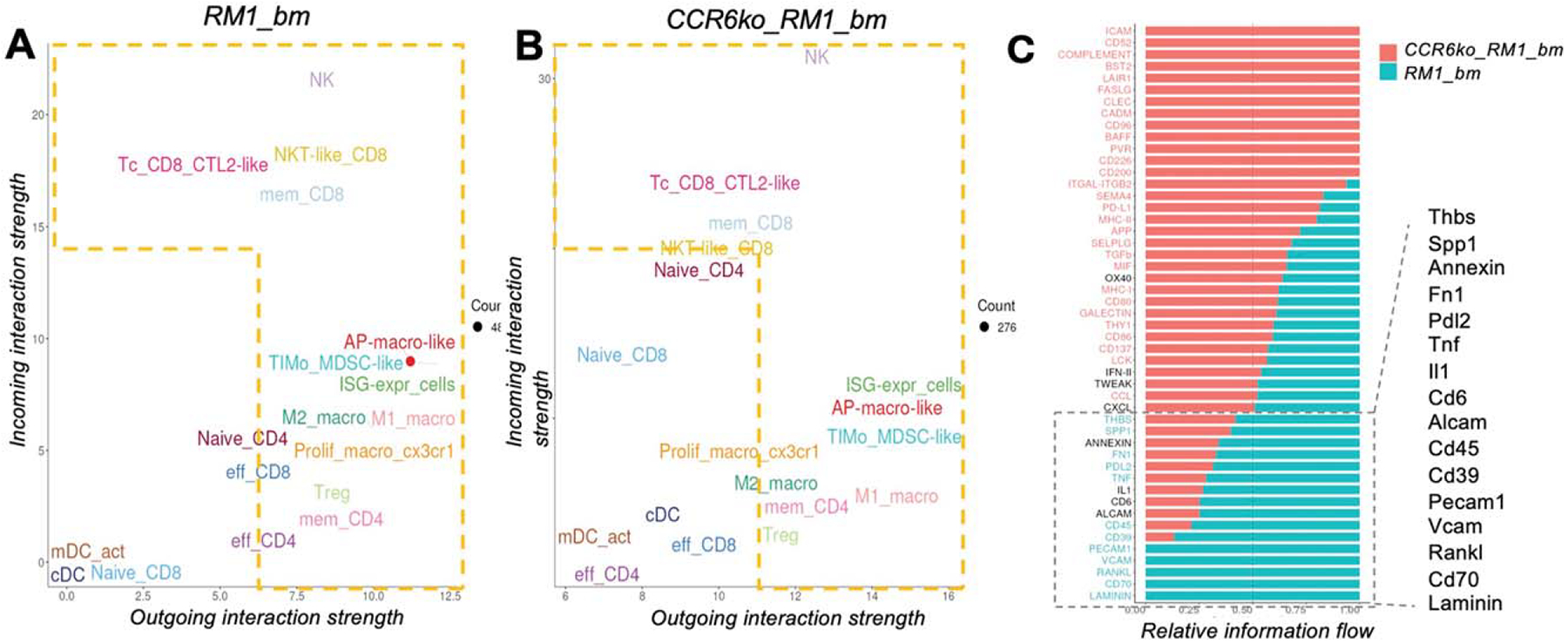
Signaling roles and patterns in tumor infiltrating cells are altered in the less malignant sample, ccr6ko_RM1_bm relative to the RM1_bm sample from a wt bone marrow environment. This representation shows the changes in signal’s incoming and outgoing interaction strength in a comparative manner between the two samples, RM1_bm (a) and CCR6ko_RM1_bm (b). (c) Relative information flow strength comparison between the two samples.

Cell–cell communication analysis revealed changes in signaling roles between tumor-infiltrating cells in RM1_bm and (ccr6ko_RM1_bm samples. In ccr6ko_RM1_bm, immune cells showed several stronger outgoing signals, including macrophage subtypes (M1, M2), Tregs, MDSC-like cells, and memory CD8+ T cells. Conversely, naïve CD4+ T cells, NK (Natural Killer) cells, and naïve CD8+ T cells shifted to an increased receiver role (Fig. 2a and b). Some populations, such as NKT-like CD8+ T cells, effector T cells, and macrophages, had reduced sender or receiver roles. Notably, conventional dendritic cells (cDCs) became stronger senders and receivers in the ccr6ko environment. Analysis of signaling changes identified upregulations of several signaling patterns in the more malignant RM1 sample (RM1_bm) relative to ccr6ko_RM1_bm: Thbs (thrombospondin), Spp1 (osteopontin), Annexin, Fn1 (fibronectin 1), Pdl2 (programmed cell death 1 ligand 2), Tnf (tumor necrosis factor), Il1 (interleukin 1), Cd6, Alcam (activated leukocyte cell adhesion molecule), Cd45, Cd39, Pecam1 (platelet endothelial cell adhesion molecule 1), Vcam (vascular cell adhesion molecule 1), Rankl (receptor activator of nuclear factor kappa-B ligand), Cd78, and Laminin (Fig. 2c).

**FIG. 3.**
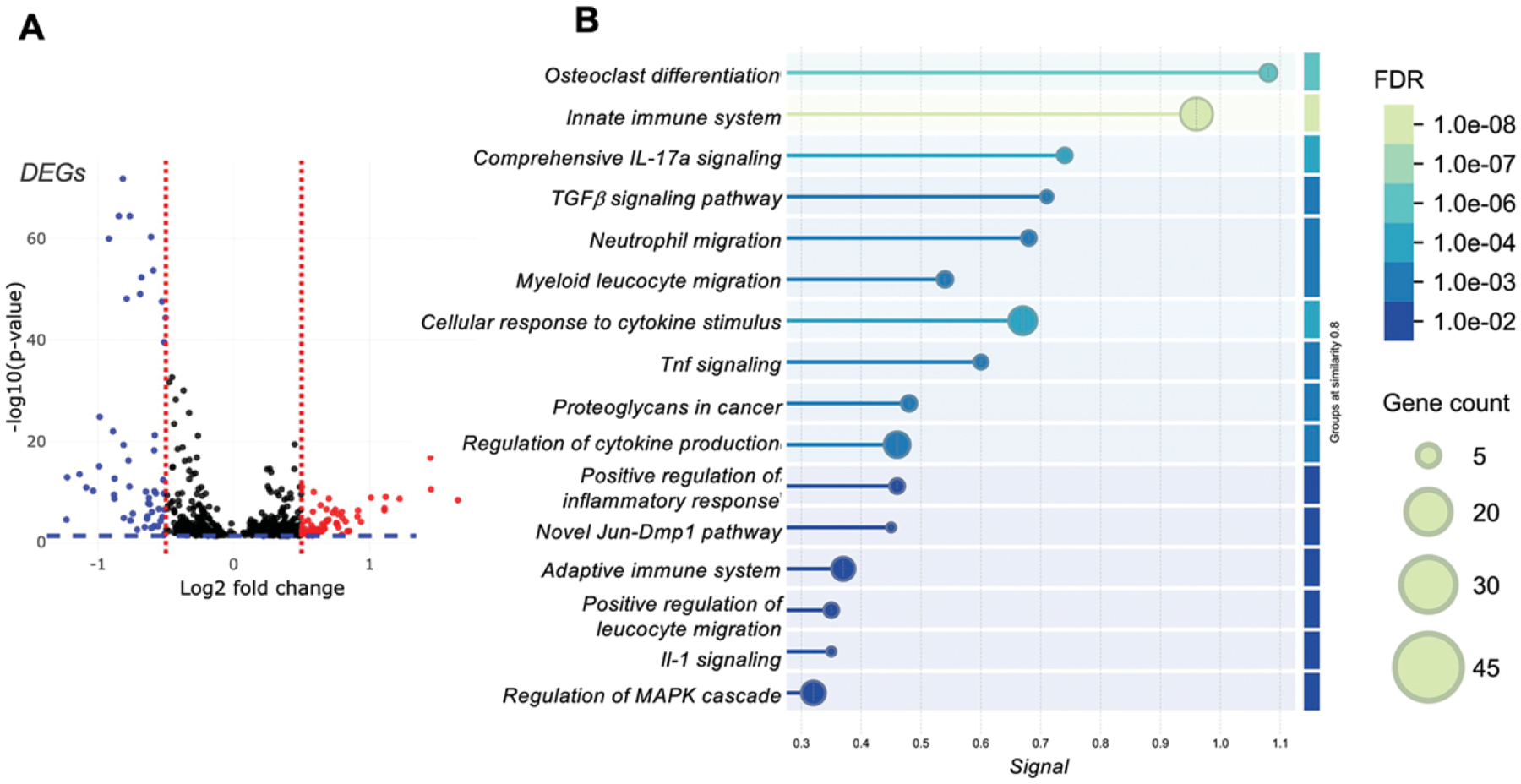
Differentially expressed genes (DEGs) upregulated in the more malignant sample (RM1_bm) relative to the less malignant sample, ccr6ko_RM1_bm, shown as (a) log2 fold change and the −log10(*p*-value). (b) Pathway enrichment analyses using STRING-DB analyses for the upregulated DEGs in RM1_bm relative to ccr6ko_RM1_bm. FDR (False Discovery Rate, color bar) and gene count shown (circle size).

Several DEGs were identified (Fig. 3a) for further analysis using STRING-DB enrichment analysis of the mouse dataset (Fig. 3b). This analysis highlighted several key pathways upregulated in the more malignant RM1 sample (the RM1_bm) relative to the less malignant sample (ccr6ko_RM1_bm), emphasizing their roles in unregulated immune regulation and tissue remodeling processes. These implicated *the innate immune system*, *IL-17 signaling*, and *TGF-β signaling*, which usually have high significance in immune modulation and inflammation processes. Additional pathways identified were *osteoclast differentiation*, *neutrophil migration*, *myeloid leukocyte migration*, and the *cellular response to cytokine stimuli*. Pathways related to *TNF signaling*, *proteoglycans in cancer*, and the *regulation of cytokine production* were also enriched, alongside the *positive regulation of inflammatory responses*, *IL-1 signaling*, and the *regulation of the MAP (Mitogen-Activated Protein) kinase cascade*. Furthermore, a *novel Jun-DMP1 pathway* and components of the *adaptive immune system* also were implicated, reflecting a complex interplay between inflammatory and immune pathways in this context.

### Cell-type-specific gene regulatory networks and their roles in immune modulation and tumor progression in RM1 prostate cancer bone metastasis

**FIG. 4.**
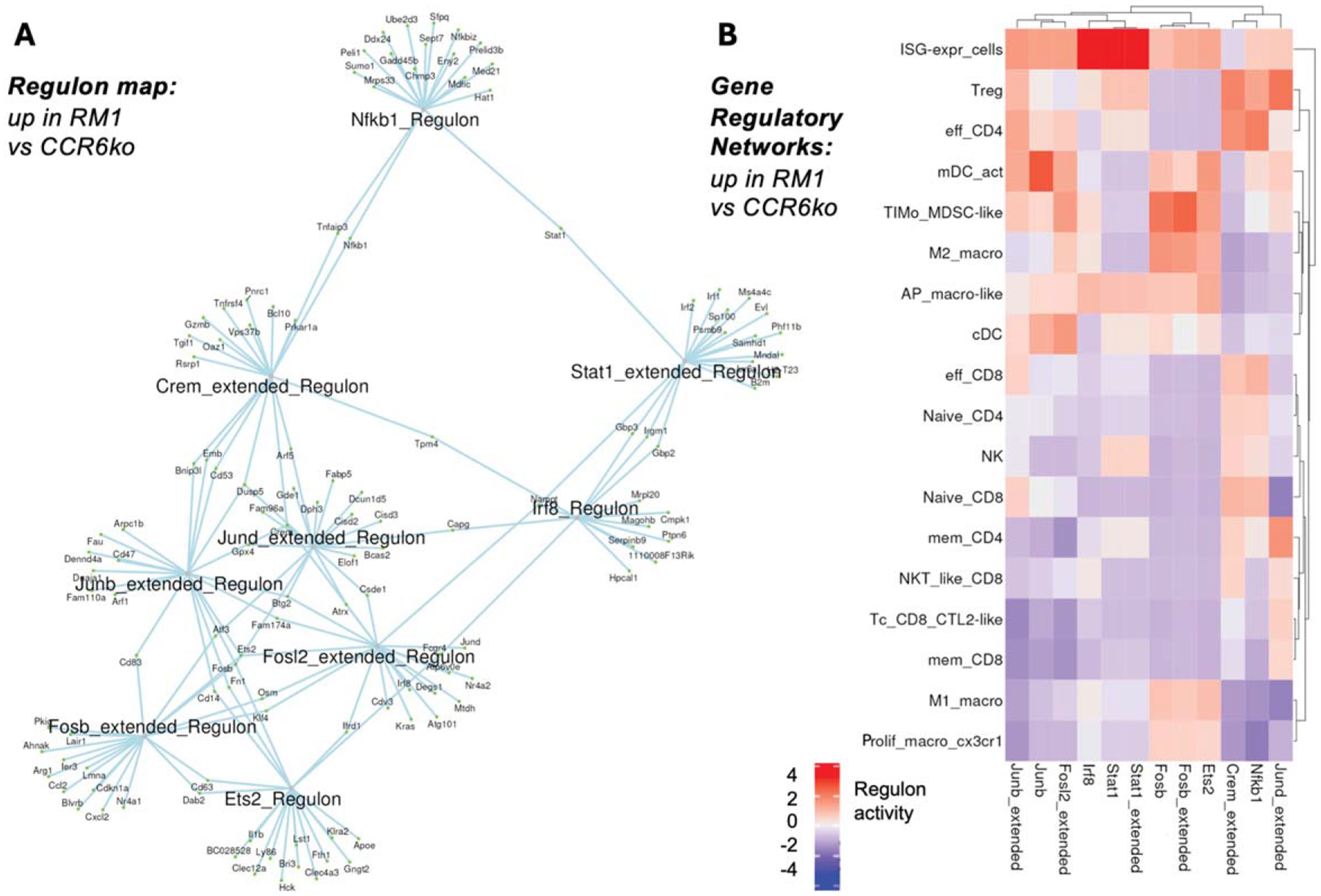
Gene regulons (a) up in RM1_bm versus ccr6ko_RM1_bm scRNAseq dataset, and similarly, (B) gene regulatory networks (color bar, regulon activity: −4 to +4 z-scores).

We identified key gene regulatory networks upregulated in the more malignant sample (RM1_bm) compared to the less malignant sample (ccr6ko_RM1_bm). Fig. 4a shows the regulon map, which included NFKB1 (nuclear factor kappa B subunit 1), STAT1 (signal transducer and activator of transcription 1), IRF8 (interferon regulatory factor 8), CREM (cAMP responsive element modulator), JUND (JunD proto-oncogene, AP-1 transcription factor subunit), JUNB (JunB proto-oncogene, AP-1 transcription factor subunit), FOSL2 (Fos-like antigen 2), FOSB (FosB proto-oncogene, AP-1 transcription factor subunit), and ETS2 (ETS proto-oncogene 2), which typically are involved in immune modulation, inflammation, and tumor progression. NFKB1 and STAT1 are critical for immune response regulation, while JUNB, FOSB, and FOSL2 are associated with cell proliferation and immune suppression in the TME. These findings suggest that these regulons may be targeted to modulate cancer growth and immune interactions in bone metastasis.

In Fig. 4b, we analyzed the expression of these regulons across immune cell subsets in the bone marrow. Interferon stimulated genes expressing (ISG-expr) cells exhibited strong STAT1 and IRF8 regulon representation, suggesting a potential involvement in immune activation. TIMo_MDSC-like cells had high FOSB representation, suggesting a potential role for this transcription factor in regulating immune suppression. mDC_act (activated dendritic cells) showed strong JUNB regulation, possibly connected with antigen presentation. Tregs and effector CD4 T cells showed correlation with NFKB1 and CREM regulons, and Tregs with JUND. Memory CD4+ T cells expressed JUND, suggesting a role for this factor in regulating memory immune responses. M2 macrophages showed strong FOSB regulation, while M1 macrophages and proliferating macrophages (CX3CR1 or C-X3-C Motif Chemokine Receptor 1) exhibited ETS2 and FOSB, suggesting potential roles for these regulators in immune modulation and inflammation. cDCs expressed both JUNB and FOSL2, supporting a potential role for these factors in regulating immune activation and T cell priming.

### Validation and comparative analysis of immune and stromal cell signaling in human and mouse bone metastasis models

We then sought to compare signaling roles in malignancy between the mouse and a human bmCRPC dataset,^7^ focusing on key immune and stromal cell populations, and their shifts in response to tumors present in the bone marrow microenvironment. Our analyses revealed both conserved and divergent changes in the signaling landscape, highlighting critical immune and stromal interactions in bmCRPC.

In both human and mouse malignancy datasets, several immune cell populations exhibited increased signaling roles. In the human dataset, Supplementary Figure 1 illustrates the UMAP of the annotated cells present in the samples with 35 clusters. In a separate analysis, NKT cells, M1 macrophages, and CD8 memory/activated cells showed increased receiver roles, while stromal cells such as vascular smooth muscle, fibroblasts, and osteoblasts (OB) were newly identified as key senders (Supplementary Figure 2). These changes aligned with findings in the mouse dataset, where NKT-like CD8 T cells and M2 macrophages showed increased receiver roles. Notably, M2 macrophages, which gained a receiving role in humans, also emerged as key players in the mouse dataset.

Substantial loss of certain signaling roles was observed in both species, particularly in adaptive immune cells. In humans, CD8 T cells (CTL2) and NK2 cells lost both receiver and sender roles, while NKT cells experienced diminished signaling capacity (Supplementary Figure 2a and b). A similar signaling loss pattern was observed in the mouse dataset, where NKT-like CD8 T cells and naive CD8 T cells lost key receiver roles. Effector CD4 T cells and cDCs also experienced a loss of both roles, indicating a broad impairment of immune signaling in these populations. Furthermore, in both species, macrophage subsets such as TIMo and M1 macrophages showed a loss of sender roles, suggesting a shift toward a more immunosuppressive or quiescent state in the TME. The transition of immune and stromal cells into less active signaling roles likely contributes to immune evasion and tumor progression. Pathway analysis of signaling changes identified several upregulations in the malignant (CRPC) sample relative to benign bone marrow samples: THBS, Laminin, IL1, FN1, SPP1, CXCL, ICAM (Intercellular Adhesion Molecule-1), and many others (Supplementary Figure 2c).

Both human and mouse datasets revealed new signaling roles for previously unannotated or poorly understood cell populations. In humans, M2 macrophages, osteoclasts (OC), and endothelial cells in the vasculature gained receiver roles, while pericytes emerged as both receivers and senders. In the mouse dataset, proliferative macrophages (Cx3cr1) were identified as gaining both receiver and sender roles, further supporting the hypothesis of a critical role for these cells in malignancy. However, it is important to note that the mouse dataset did not capture vascular cells or fibroblasts, as it was limited to CD45-negative cells. Consequently, vascular remodeling roles, such as those observed in human endothelial cells and pericytes, were not represented in the mouse dataset.

Furthermore, since pathways can be conserved across species, we sought to validate the presence of the differentially regulated pathways or gene networks in this human scRNAseq dataset. This helps establish that the underlying biological processes identified in the mouse model may be relevant to humans.

Several DEGs were identified (Supplementary Figure 3a) for further analysis using STRING-DB enrichment analysis of the human dataset (Supplementary Figure 3b). STRING-DB analysis identified several key pathways, including the *innate immune system, signaling by NOTCH, adaptive immune system, MAPK (Mitogen-activated Protein Kinase) family cascades, MHC II (Major Histocompatibility Complex) protein complex binding, response to cytokines, transcription factor AP-1, signaling by the TGF-β (Transforming Growth Factor beta) receptor complex, leukocyte migration, positive regulation of myeloid cell differentiation, osteoblast differentiation*, and *positive regulation of endothelial cell migration*.

The gene networks identified in the human bmCRPC dataset (Supplementary Figure 4a) showed both similarities and differences compared to the mouse dataset. In the human dataset, key regulators included HDAC2 (histone deacetylase 2), CREM (cAMP responsive element modulator), YY1 (Yin Yang 1), REL (v-rel avian reticuloendotheliosis viral oncogene homolog), XBP1 (X-box binding protein 1), KLF6 (Krüppel-like factor 6), ATF4 (activating transcription factor 4), ETS1 (ETS proto-oncogene 1), and JUN/JUNB/JUND/FOSB (members of the AP-1 transcription factor complex), forming a central hub in the network. These factors are involved in various cellular processes, including stress response, inflammation, and tumor progression. The presence of these JUN/FOS regulons as central nodes suggests a strong role in mediating the transcriptional response to the CRPC tumor in the bone marrow microenvironment. Central AP-1 (JUN/FOS) and ETS contributions appear to regulate the TME, although some other transcriptional regulators also were prominent. In Supplementary Figure 4b, we analyzed the expression of these regulons across immune cell subsets in the bone marrow from the human dataset. ETS1 showed high expression in naïve CD4+ T cells, CD8+ memory T cells, CD4 activated T cells, CD8+ cytotoxic-like T cells, NKT cells, and CD4+ memory T cells. JUN/FOS regulons were strongly upregulated in OB, vascular smooth muscle cells, ISG-expressing cells, pericytes, endothelial cells, and vascular remodeling cells. Expression was also observed in M1 macrophages, OC, and plasma cells, although plasma cells and plasmacytoid dendritic cells exhibited more evident XBP1- and ATF4-enriched regulons instead. CREM regulon was highly represented in OB, endothelial cells, M1 macrophages, NKT cells, and CD4+ memory T cells. JUND peaked in M1 macrophages and OB, while the HDAC2 regulon was highest in proliferating erythroid cells, hematopoietic stem cells, pro-B cells, MDSCs, activated myeloid dendritic cells, OB, pericytes, and cancer cells. Overall, our findings indicated a dominant role for ETS and JUN/FOS transcriptional programs in this human bone marrow dataset, which were similar to the central types of transcription factors identified in the mouse scRNAseq dataset.

## Discussion

Prostate cancer interaction with the bone microenvironment is driven by a unique interplay between tumor cells, immune populations, and bone cells. By leveraging scRNAseq technology, we identified distinct cellular populations and signaling networks that illustrated the complexity of tumor–immune–stromal interactions within the bone niche. Our study highlights the critical role of CCR6 and its associated pathways in shaping the TME of prostate cancer bone metastases. These findings can be highly useful in proposing novel therapeutic avenues. CCR6 inhibition strategies (genetic or chemical/biological) may work toward reprogramming the TME toward a more immunologically active state. Since CCR6 can help in the recruitment of pro-inflammatory cells and may impact macrophage polarization, CCR6 blockade could potentially prime prostate cancer bone metastases to respond better to existing immunemodulating by reducing immune suppression and better supporting antitumor immunity approaches.

Our scRNAseq analysis specifically revealed increased signaling from macrophages (M1 and M2), Tregs, and MDSC-like cells, along with stronger incoming signals to naïve T cells, CD8+ T cells, NK cells, and cDCs when the bone marrow had CCR6 knocked-down. These findings suggest that CCR6 contributes to a tumor-supportive immune microenvironment by promoting the recruitment of immunesuppressive cells while limiting antitumor immune responses from effector T and NK cells. This would align with the notion that CCR6 signaling promotes immune evasion mechanisms, which are critical for tumor progression. Moreover, our DEG analysis provided additional insights into the molecular changes associated with CCR6 deficiency. DEGs in the more malignant sample (RM1_bm) were enriched in several pathways related to immune response, inflammation, and tumor progression. These included *immune system dysregulation, IL-17A* and *TGFβ signaling, neutrophil and myeloid leukocyte migration, cytokine response*, and the *regulation of inflammatory responses*. Additionally, pathways involved in osteoclast differentiation and bone remodeling, such as *proteoglycans*, were identified, suggesting a role in malignancy-related bone changes. Altered signal transduction pathways, including *transcription factor signaling, MAPK regulation*, and *cytokine production*, may further contribute to the TME. The involvement of the *adaptive immune system* and a *Jun-Dmp1 pathway* points to broader immune and molecular changes that could influence metastasis and therapeutic resistance. These findings suggest that CCR6 deficiency disrupts immune communication and modifies tumor-stromal interactions, fostering a protumorigenic environment.

A comparative analysis of mouse and human datasets emphasized the conserved nature of immune modulation in malignant samples, i.e., bmCRPC. In the human dataset, ETS1, FOSB, JUN family members, and CREM were highly expressed across immune and stromal populations in the malignant samples relative to benign bone marrow. These factors are often implicated in immune activation, stromal remodeling, and macrophage polarization.^12,13^ For example, ETS1 showed enrichment across T cell subtypes, highlighting its central role in T cell regulation. Similarly, JUN/FOS family members were strongly enriched in stromal cells, including OB, endothelial cells, and pericytes, suggesting their involvement in inflammatory responses and vascular remodeling. CREM, primarily enriched in OB and NKT cells, reinforces its dual role in stromal–immune interactions. In contrast, HDAC2 enrichment was notable in hematopoietic and progenitor populations, suggesting its role in regulating immune suppression. These transcription factors present attractive therapeutic targets for reshaping the immune microenvironment and enhancing antitumor responses. Despite some species-specific differences, the conserved roles of regulators from the families of ETS and JUN/FOS transcription factors may establish a solid foundation for translational strategies targeting immune evasion in bmCRPC.

Key immune cell populations, including M2 macrophages, NKT or NKT-like CD8 T cells, and proliferative macrophages (CX3CR1), consistently exhibited shifts in both human and mouse datasets from the more malignant samples. M2 macrophages displayed increased receiver roles, implicating their enhanced involvement in immunosuppressive signaling pathways. This shift from pro-inflammatory M1 to immunosuppressive M2 macrophages highlights a critical mechanism for tumor immune evasion.^14^ Targeting macrophage polarization or promoting M1 functionality could provide effective therapeutic strategies to reverse this imbalance.

In both datasets, STRING-DB enrichment analysis revealed conserved pathways, including *IL-17 signaling*, *TGF-β signaling*, and *innate immune regulation*. These pathways are centered on immune modulation, stromal remodeling, and inflammatory processes in cancer. In the human dataset, additional enriched pathways included *adaptive immune responses, leukocyte migration*, and *endothelial cell regulation*, which may reflect a broader spectrum of immune and stromal interactions. The upregulation of key extracellular matrix components (e.g., laminin, FN1, and SPP1) and angiogenic factors (e.g., Vascular Endothelial Growth Factor or VEGF and Vascular Cell Adhesion Molecule-1 or VCAM) in both datasets further highlights the shared mechanisms driving tumor-stroma crosstalk.

Despite the overall similarities between the mouse and human datasets, our study reveals certain species-specific differences, which are common limitations when attempting to translate findings from preclinical models to human disease. For instance, human bmCRPC samples showed a broader upregulation of genes associated with angiogenesis and immune modulation, such as Integrin Beta-2 or ITGB2, Angiopoietin-like proteins or ANGPTL, and CXCL family members. These genes are implicated in endothelial cell migration, angiogenesis, and immune cell recruitment, suggesting that the human TME in bone metastasis may involve more extensive vascular remodeling and immune cell infiltration compared to the murine model. The upregulation of these angiogenic and immune-related factors in human bmCRPC may reflect a more complex TME, where both the vascular and immune components work in concert to promote tumor progression and immune evasion. In contrast, the mouse dataset uniquely highlighted markers like Cd70 and Rankl, which are specifically involved in immune cell recruitment and activation. Cd70, for example, is a ligand for Cd27, a costimulatory receptor on T cells, and its increased expression in the mouse model suggests a greater reliance on T cell-mediated immunity in driving the metastatic process. Rankl, a key player in osteoclast differentiation and bone resorption, was also upregulated in the mouse model, indicating that the murine TME may be more focused on bone degradation mechanisms as a means of supporting tumor growth and metastasis. These differences suggest that while certain immune pathways may be conserved across species, there are distinct immune responses and tumor–stromal interactions in mouse and human models of prostate cancer bone metastasis. However, it is important to note that some of these differences may be attributed to the mouse dataset having been specifically focused on immune cells and excluded non-immune CD45-negative cells, whereas the human dataset also contained many other clusters of significant impact, such as vascular, bone cells, progenitors, etc.

The species-specific variations observed highlight the importance of considering the inherent biological differences between preclinical models and human disease when developing new therapeutic strategies. Translating findings from mouse models to human treatments requires a nuanced understanding of these differences, as what works in one species may not always translate directly to another. Our results emphasize the need for additional validation in human models in future studies, such as patient-derived xenografts or organoid systems, to confirm the relevance of these findings to human prostate cancer bone metastasis. Further studies should focus on exploring how the molecular mechanisms behind these species-specific differences may impact therapeutic outcomes, and the RM1-based mouse models as well as some other more recently described osteogenic cell syngeneic mouse models^11,15^ appear to be highly promising tools for achieving some of these goals.

Therapeutic positioning of our findings may include leveraging CCR6 inhibition toward synergistic enhancement by potentially combining it with immune checkpoint inhibitors, such as anti-PD-1 (Programmed Cell Death Protein 1) or anti-PD-L1 (Programmed Death-Ligand 1), which may further boost T cell activation and antitumor immunity. Additionally, targeting the IL-6/IL-6R and TGFβ signaling pathways could help alleviate the immune suppression typically seen in the TME, promoting a more favorable immune environment. Given the upregulation of IL-17 signaling, therapies targeting this cytokine or expressing an antagonist, like IL-27, could further help in reducing inflammation and tumor progression. Modulating the Th17/Treg axis, either through IL-17 inhibitors or Treg-depleting agents, offers another promising strategy to rebalance immune responses and improve therapeutic outcomes. Potentially, our findings point to the promise of also addressing tumor–stroma interactions in bone metastasis. Inhibiting angiogenic factors like VEGF, extracellular matrix components (e.g., laminin, FN1, SPP1), or signaling pathways involved in stromal remodeling (e.g., MAPK activation and proteoglycans) could reduce tumor spread, decrease bone degradation, and limit immune evasion. These strategies, when combined with immune checkpoint inhibitors and cytoskine modulation, could offer a comprehensive approach to targeting both the immune microenvironment and tumor–stroma interactions in prostate cancer bone metastasis.

In summary, this study offers novel insights into the role of CCR6 in prostate cancer bone metastasis and presents a foundation for future therapeutic approaches. By combining CCR6 inhibition with immune-modulatory therapies and strategies targeting tumor-stroma interactions, more effective, personalized treatments could be developed that address both immune suppression and the complexity of the TME.

## Supplementary Material

S3

S2

S4

S1_legends

S1
