# Supplementary figures and images for: Single-Cell RNA Sequencing Identifies CCR6-Driven Immune Landscape Changes in RM1 Prostate Cancer Bone Metastasis
Source: DNA Cell Biol Rep. Author manuscript; Available in PMC 2026 Apr 18. (PMC13089933; doi:10.1089/dcbr.2025.0001)

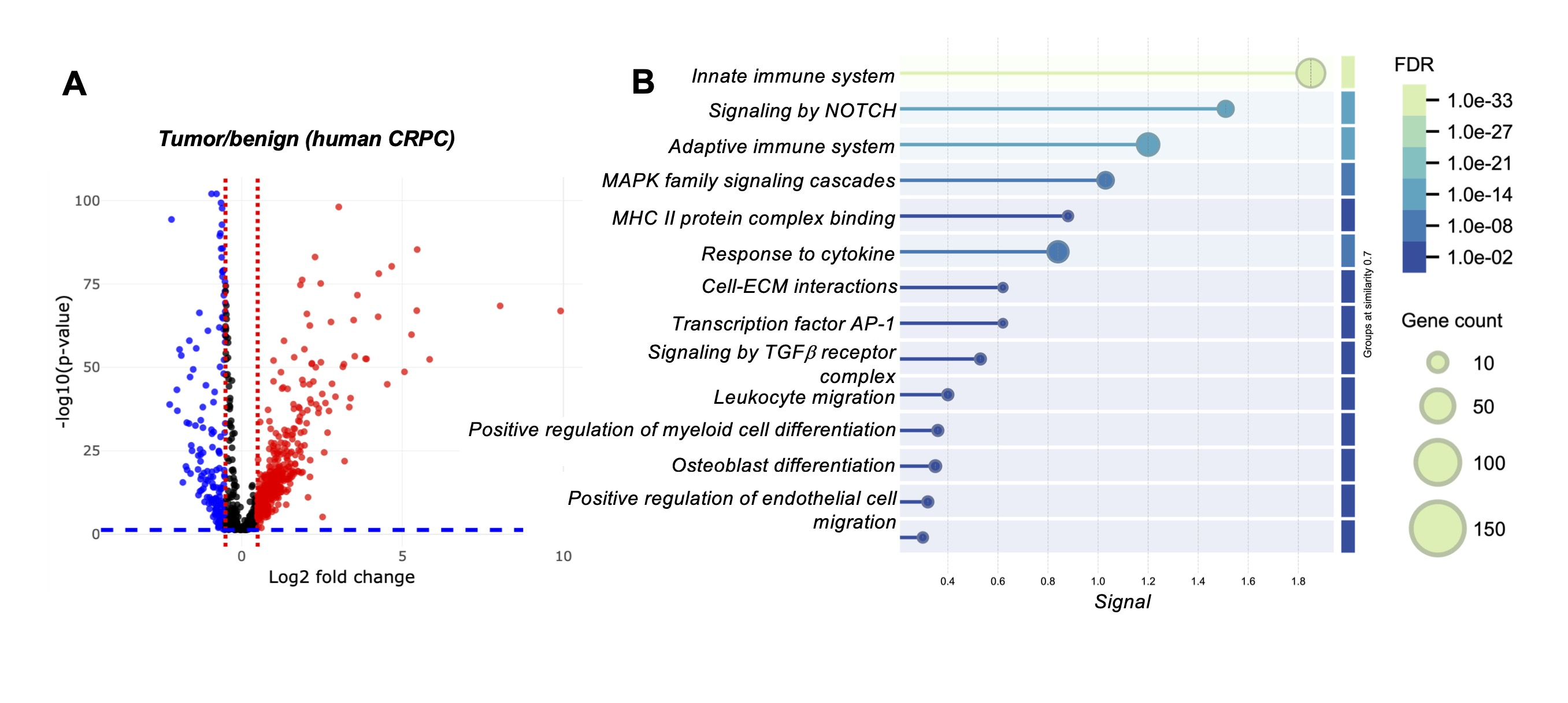

Supplement: S3 [file NIHMS2162479-supplement-S3.tif]

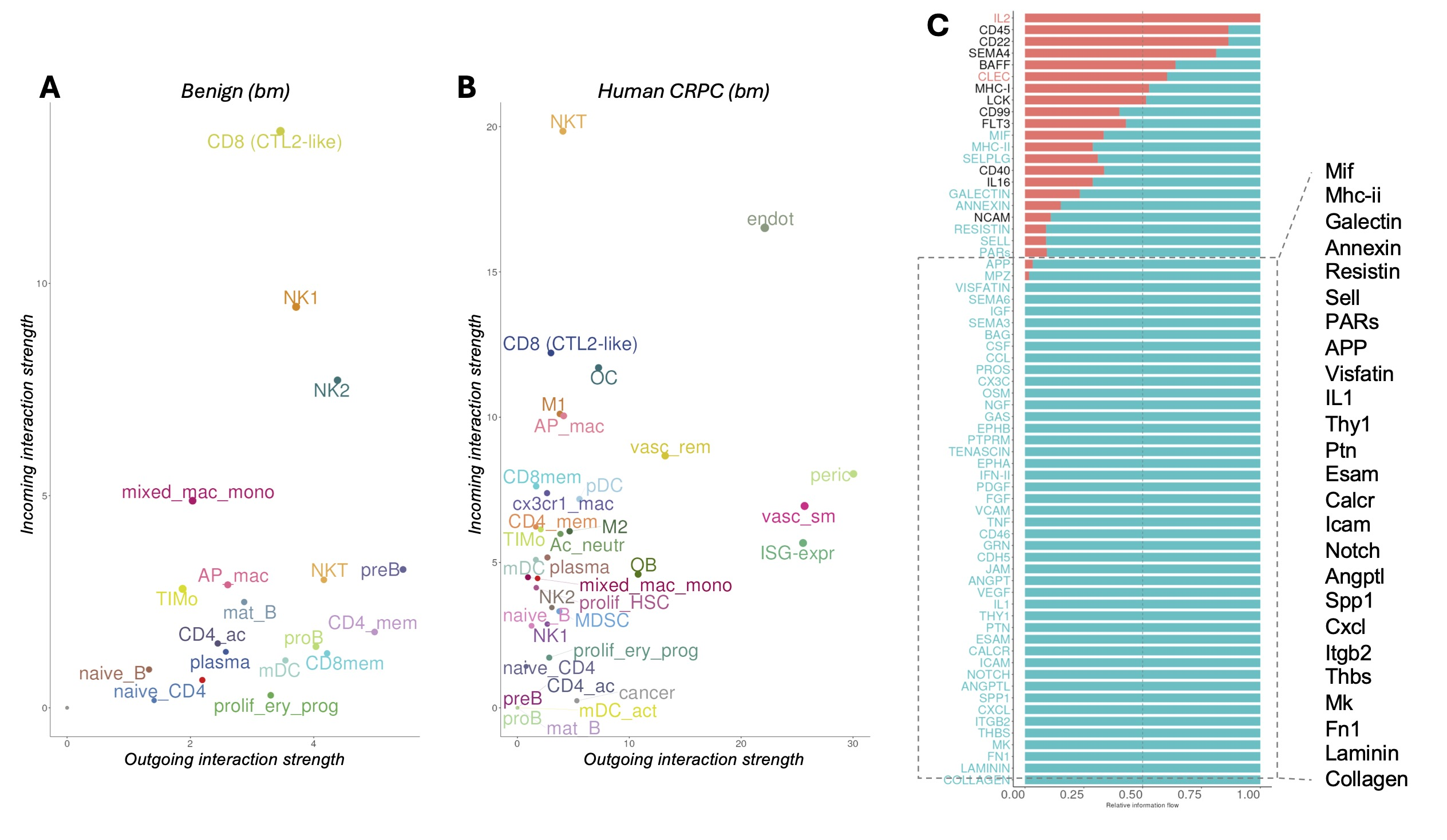

Supplement: S2 [file NIHMS2162479-supplement-S2.tif]

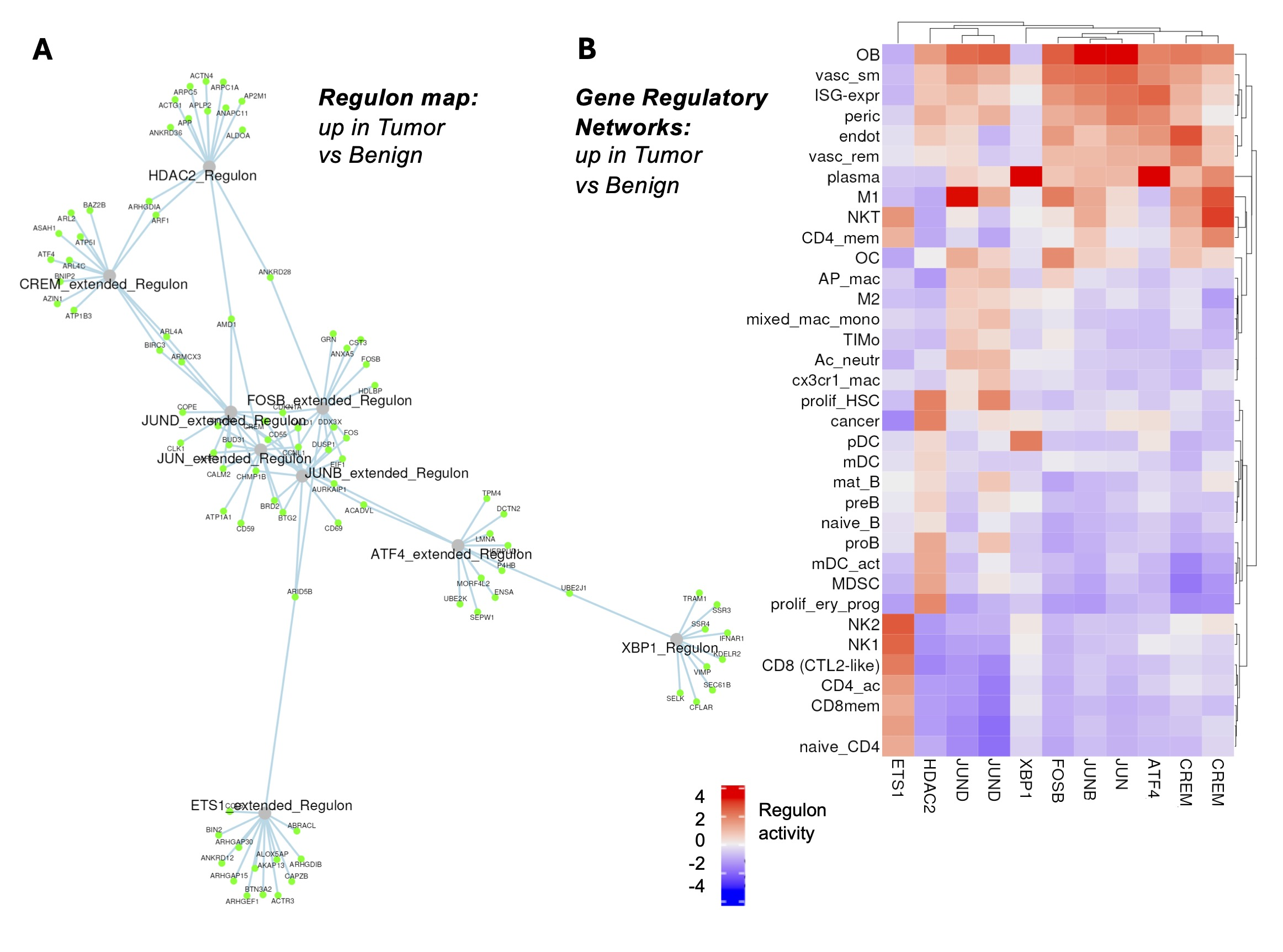

Supplement: S4 [file NIHMS2162479-supplement-S4.tif]

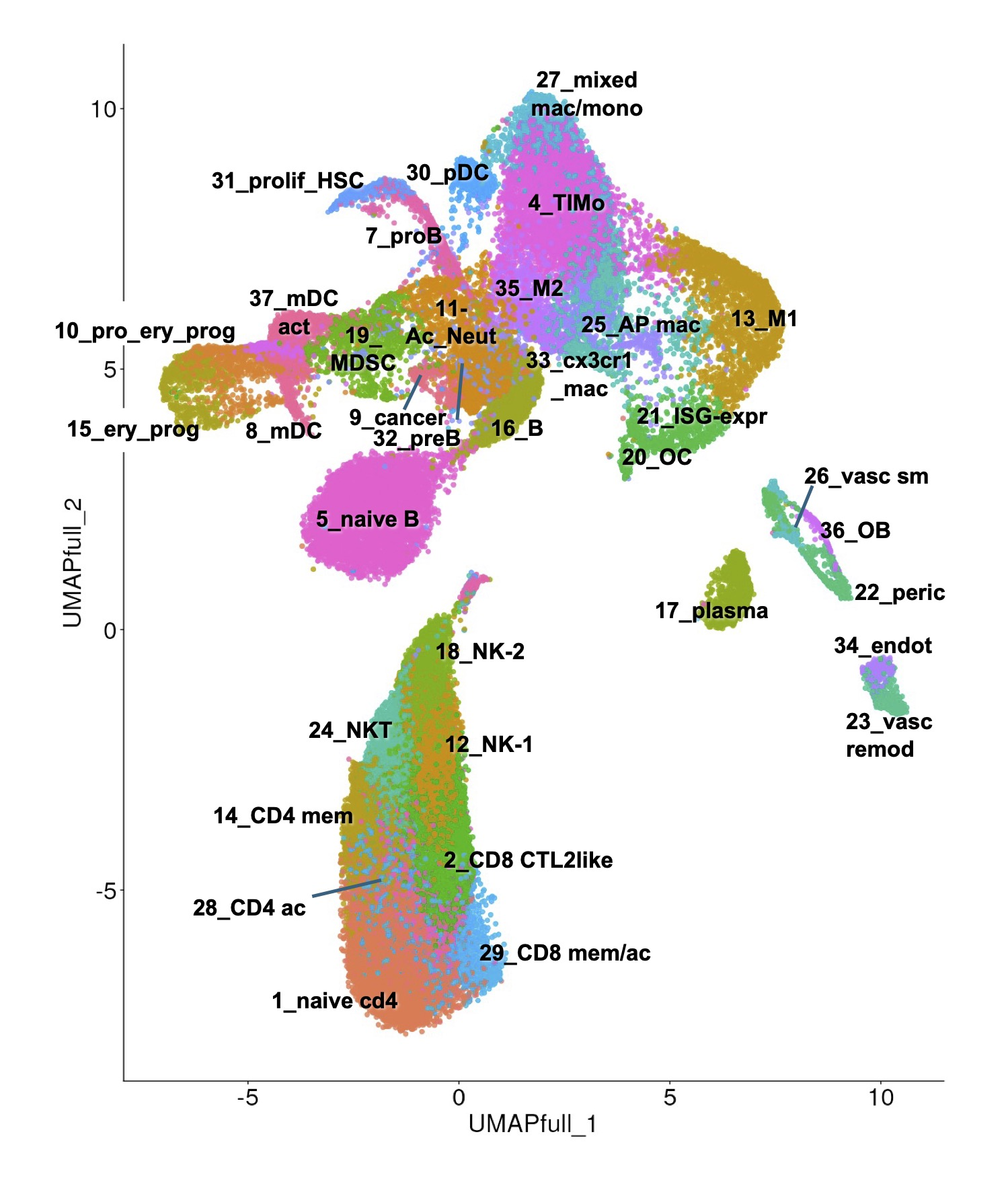

Supplement: S1 [file NIHMS2162479-supplement-S1.tif]
