# Supplementary material for: Single-Cell RNA Sequencing Identifies CCR6-Driven Immune Landscape Changes in RM1 Prostate Cancer Bone Metastasis
Source: DNA Cell Biol Rep. Author manuscript; Available in PMC 2026 Apr 18. (PMC13089933; doi:10.1089/dcbr.2025.0001)
Supplement: S1_legends [file NIHMS2162479-supplement-S1_legends.docx]

**Suppl. Fig. 1. Cell types (human) identified in the scRNAseq analysis using Icarus after integration with Harmony for tumor and benign bone marrow samples.** The UMAP projections are displayed on the UMAPfull_1 and UMAP_2 axes, showing clustering patterns of cell types based on their gene expression profiles. This integration allows for a comprehensive comparison of cell-type distribution between the tumor and benign bone marrow microenvironments.

**Suppl. Fig. 2. Cell-cell communication analyses (human dataset).** (A) Interaction strength patterns versus outgoing interaction strength, illustrating shifts in cell population signaling between benign and bmCRPC samples. (B) Bar chart highlighting key differences in signaling within the bmCRPC sample, with upregulated cell communication signals identified.

**Suppl. Fig. 3.** Differentially expressed genes (DEGs) upregulated in the human dataset by comparing the malignant samples (CRPC) relative to the benign bone marrow samples, shown as (A) log2 fold change and the -log10(p-value). (B) Pathway enrichment analyses using STRING-DB analyses for the upregulated DEGs in CRPC relative to benign samples. FDR (color bar), and gene count shown (circle size).

**Suppl. Fig. 4**. Gene Regulons (A) up in tumor (CRPC) versus benign samples in the human scRNAseq dataset, and similarly, (B) Gene regulatory networks (color bar, regulon activity: -4 to +4 z-scores).
